# Optimization and Deoptimization of Codons in SARS‐CoV‐2 and Related Implications for Vaccine Development

**DOI:** 10.1002/advs.202205445

**Published:** 2023-06-02

**Authors:** Xinkai Wu, Ke‐jia Shan, Fuwen Zan, Xiaolu Tang, Zhaohui Qian, Jian Lu

**Affiliations:** ^1^ State Key Laboratory of Protein and Plant Gene Research Center for Bioinformatics School of Life Sciences Peking University Beijing 100871 China; ^2^ NHC Key Laboratory of Systems Biology of Pathogens Institute of Pathogen Biology Chinese Academy of Medical Sciences and Peking Union Medical College Beijing 100176 China

**Keywords:** codon optimization, codon usage bias, SARS‐CoV‐2, spike protein, synonymous mutations, vaccine design

## Abstract

The spread of coronavirus disease 2019 (COVID‐19), caused by severe respiratory syndrome coronavirus 2 (SARS‐CoV‐2), has progressed into a global pandemic. To date, thousands of genetic variants have been identified among SARS‐CoV‐2 isolates collected from patients. Sequence analysis reveals that the codon adaptation index (CAI) values of viral sequences have decreased over time but with occasional fluctuations. Through evolution modeling, it is found that this phenomenon may result from the virus's mutation preference during transmission. Using dual‐luciferase assays, it is further discovered that the deoptimization of codons in the viral sequence may weaken protein expression during virus evolution, indicating that codon usage may play an important role in virus fitness. Finally, given the importance of codon usage in protein expression and particularly for mRNA vaccines, it is designed several codon‐optimized Omicron BA.2.12.1, BA.4/5, and XBB.1.5 spike mRNA vaccine candidates and experimentally validated their high levels of expression. This study highlights the importance of codon usage in virus evolution and provides guidelines for codon optimization in mRNA and DNA vaccine development.

## Graphical abstract

This study reveals that SARS‐CoV‐2 tends to use nonoptimal human codons, displaying a decreasing codon adaptation index over time. Experiments show that synonymous mutations significantly impact viral protein expression, potentially affecting SARS‐CoV‐2 fitness. Researchers design and validate codon‐optimized spike mRNA vaccine candidates for Omicron BA.2.12.1, BA.4/5, and XBB.1.5 variants, which could contribute to more effective vaccines against SARS‐CoV‐2 variants.

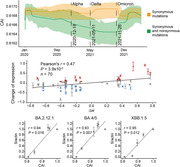

## 1 Introduction

Amino acids serve as the fundamental building blocks of proteins. Eighteen out of the twenty amino acids are encoded by at least two synonymous codons in all domains of life, with methionine and tryptophan as the two exceptions. The synonymous codons for an amino acid are often used at different frequencies because of variability in the host cell's tRNA supply, which is referred to as “codon usage bias” (CUB). CUB is observed in the most of organisms to varying degrees.^[^ ^1^ ^]^ Because of degeneracy of the genetic code, synonymous mutations in protein‐coding regions are frequently considered neutral or almost neutral since they do not alter the protein sequence.^[^ ^2^ ^]^ However, accumulating evidence suggests that synonymous mutations are not entirely neutral in evolution.^[^ ^3^ ^]^ By altering translation efficiency,^[^ ^4^ ^]^ mRNA stability,^[^ ^5^ ^]^ and peptide conformation,^[^ ^6^ ^]^ synonymous mutations may impact protein expression and function and, eventually, the fitness of the organism.^[^ ^3a^ ^]^

Viruses typically rely on the cellular machinery of their host organisms for biological processes such as translation. Viruses usually exhibit a modest level of CUB, presumably due to mutational pressure.^[^ ^7^ ^]^ It has been speculated that viruses with low and inefficient codon usage could adapt to various host species with different codon usage preferences.^[^ ^7^, ^8^ ^]^ Factors in the host cellular environment frequently attack RNA virus genomes, and such attacks can induce specific types of mutations.^[^ ^9^ ^]^ For instance, apolipoprotein B mRNA editing enzymcatalytic polypeptide‐like (APOBEC) family enzymes could produce C>U mutations in viral RNA genomes, while adenosine deaminase acting on RNA (ADAR) family enzymes can produce A>G mutations.^[^ ^10^ ^]^ It was demonstrated that codon usage patterns in RNA viruses might be primarily influenced by mutational pressure.^[^ ^7^, ^11^ ^]^ Natural selection may also play an important role in modulating viral CUB, although its effectiveness may vary among viruses. For instance, natural selection has significantly influenced how codons are used in coronaviruses.^[^ ^12^ ^]^ Furthermore, too much similarity in CUB between viruses and hosts negatively impacts both the viruses and the hosts.^[^ ^3b^ ^]^

SARS‐CoV‐2, the etiologic agent of coronavirus disease 2019 (COVID‐19), has a codon usage pattern different from that of humans and bats.^[^ ^8^, ^13^ ^]^ Studies have revealed that as the pandemic spread, the codon usage pattern of SARS‐CoV‐2 diverged further from that of its human hosts rather than evolving to use more optimized codons.^[^ ^13^, ^14^ ^]^ Despite these discoveries, the evolutionary driving forces underlying the optimization and deoptimization of codons in SARS‐CoV‐2 are poorly understood. Some studies suggest that natural selection is the main force shaping the codon usage patterns in SARS‐CoV‐2,^[^ ^15^ ^]^ while others argue that mutational pressure is the primary factor determining the codon usage of this virus and that natural selection plays only a minor role.^[^ ^16^ ^]^ Additionally, some studies suggest that the codon usage of SARS‐CoV‐2 is influenced by both mutational bias and natural selection.^[^ ^13b,c^ ^]^ Therefore, significant gaps remain in our understanding of the evolutionary principles underlying CUB in SARS‐CoV‐2. Furthermore, experimental evidence that links codon usage to the translational efficiency of SARS‐CoV‐2 genes is still lacking.

Here, we analyzed 9164789 high‐quality SARS‐CoV‐2 genomes for codon usage profiling. We found that the deoptimization of codons throughout the evolution of SARS‐CoV‐2 genomes is primarily influenced by amino acid changes, while synonymous mutations had little effect. We identified a strong bias toward C>U substitutions, primarily driven by the mutational bias of the host nucleotide editing system. It is possible that some of these substitutions were also influenced by natural selection. We showed experimentally that codon optimization of SARS‐CoV‐2 improves protein translation in human cells. Finally, we showed that optimizing the *Spike* gene codon usage of SARS‐CoV‐2 variants has important implications for vaccine design.

## 2 Results

### 2.1 Codon Deoptimization in the Continuing Evolution of SARS‐CoV‐2 is mainly caused by Amino Acid Changes

**Figure 1:**
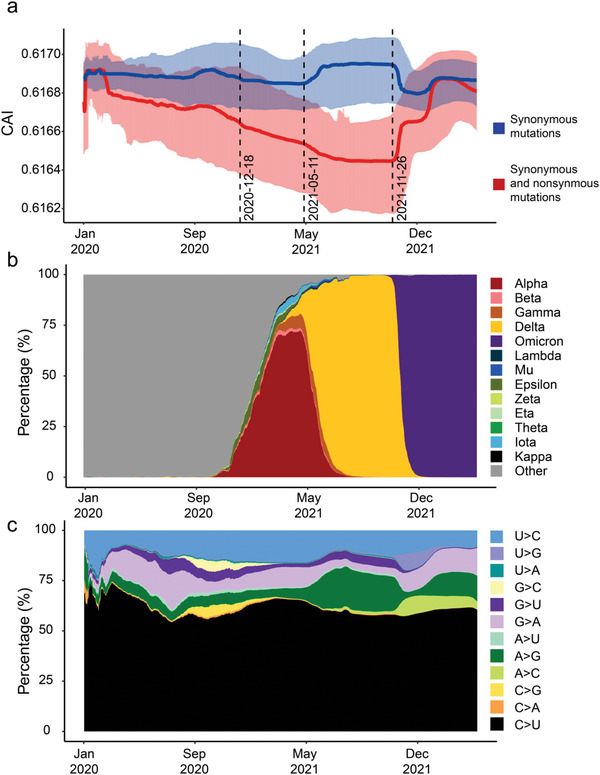
The evolutionary trends in codon adaptation index (CAI, major viral lineages, and synonymous mutation types during SARS‐CoV‐2 evolution. a) The CAI changes in SARS‐CoV‐2 caused by both synonymous and nonsynonymous (the solid red line and shadow) or synonymous mutations only (the solid blue line and shadow) over time. The red and blue solid lines indicate the median CAI, and the red and blue shadows indicate the 95% interval of CAI in a 14‐day sliding window with a one‐day step. The black dashed lines indicate when the World Health Organization defined the Alpha, Delta, and Omicron lineages as variants of concern (VOCs). b) Prevalence of VOCs and variants of interest (VOIs) over time. The proportion of variants of SARS‐CoV‐2 in a 14‐day sliding window with a one‐day step. c) The proportions of synonymous mutations of different substitutional types in SARS‐CoV‐2 in a 14‐day sliding window with a one‐day step.

We downloaded 9164789 high‐quality SARS‐CoV‐2 genomes from the Global Initiative on Sharing All Influenza Data (GISAID, https://www.gisaid.org; as of June 18, 2022) and calculated the codon adaptation index (CAI) of the concatenated coding sequences of each genome. The CAI values ranged from 0.6154 to 0.6192, with a median value of 0.6166 and 2.5th and 97.5th percentiles of 0.6162 and 0.6169, respectively. To decipher the evolutionary trend in CAI as the pandemic developed, we compared the CAI values of the SARS‐CoV‐2 genomes collected on different dates. Our observations were consistent with previous findings that CAI decreased during the first four ^[^ ^14^ ^]^ and 18 months ^[^ ^13f^ ^]^ of SARS‐CoV‐2 evolution in humans (**Figure** **1**a, the red lines). However, we observed a turning point in CAI around November 26, 2021, ≈23 months after the beginning of the COVID‐19 pandemic. Before this time point, the CAI value decreased as the Alpha variant (B.1.1.7) became the predominant lineage and then dropped further as the Delta variant (B.1.617.2) replaced Alpha and other variants of concern (VOCs) or variants of interest (VOIs). After November 26, 2021, the Omicron variant (B.1.1.529) rapidly replaced Delta to become the predominant SARS‐CoV‐2 variant, and correspondingly, the CAI value of SARS‐CoV‐2 increased (Figure 1a,b).

As the pandemic has progressed, positive selection has driven many amino acid substitutions in the SARS‐CoV‐2 genome.^[^ ^17^ ^]^ In particular, the Alpha, Delta, and Omicron variants differ from the reference genome (NC_04 5512) by at least 17, 26, and 31 amino acids, respectively (retrieved from Outbreak.info project, https://outbreak.info/). To examine whether the decrease or increase in CAI values was attributable to amino acid changes in the VOCs/VOIs, we excluded nonsynonymous changes that alter amino acids and calculated the CAI value of each genome (that is, we considered only synonymous changes in the CAI analysis). Interestingly, we found that the CAI value fluctuates slightly in a narrow range between 0.6167 and 0.6171 (Figure 1a, the blue lines). In addition, we examined the evolutionary trends of CAI values in different regions of the SARS‐CoV‐2 genomes. While the *ORF1ab* and *S* genes contained sufficient mutations for analysis, the nine remaining individual genes (*ORF3a*, *E*, *M*, *ORF6*, *ORF7a*, *ORF7b*, *ORF8*, *N*, and *ORF10*) were generally short and did not yield informative results. Consequently, we combined the remaining nine genes for the CAI analysis. We also observed similar trends in CAI shaped by synonymous and nonsynonymous mutations, although the extent of the patterns varied (Figure S1, Supporting Information). In other words, synonymous changes only slightly affected the CAI values of SARS‐CoV‐2 throughout the pandemic, and the relatively large decreases or increases in CAI between dominant variants mainly resulted from nonsynonymous substitutions in SARS‐CoV‐2. We hypothesize that the overall changes in CAI value among SARS‐CoV‐2 variants may be coincidental byproducts of amino acid changes that may affect the replication, transmission, or immune evasion of SARS‐CoV‐2.

### 2.2 Codon Deoptimization by C>U Substitution Bias in SARS‐CoV‐2

Apolipoprotein B mRNA editing enzymcatalytic polypeptide‐like (APOBEC) APOBEC RNA editing enzymes frequently induce C>U mutations in the single‐stranded viral RNA molecules,^[^ ^18^ ^]^ causing excessive C>U substitutions in SARS‐CoV‐2 genomes.^[^ ^18^, ^19^ ^]^ Notably, the C>U substitutions, which account for ≈60% of all the nucleotide changes accumulated in the synonymous sites in the SARS‐CoV‐2 genomes, remained dominant over time despite the frequent replacement of viral lineages (Figure 1c).

**Figure 2:**
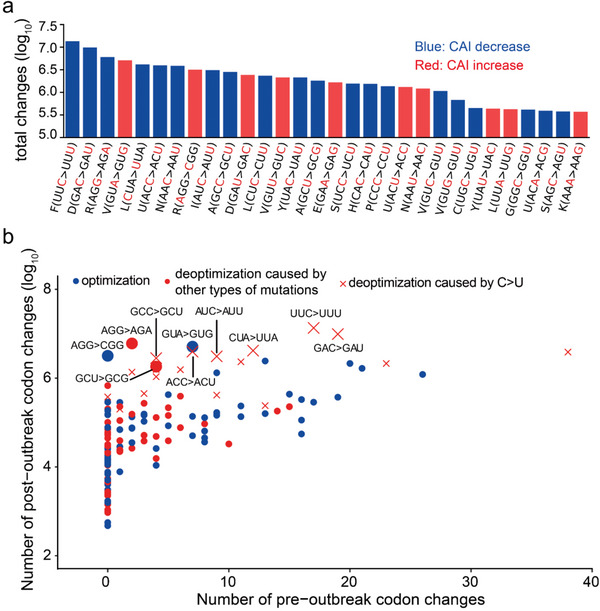
Deoptimization of codons by C>U substitutional bias in SARS‐CoV‐2. a) Types of the top 30 most frequently observed codon substitutions. Red denotes an increase in the sequence's CAI, whereas blue suggests a decrease because of the substitution. b) The number of pre‐ or post‐outbreak synonymous codon changes. The *x*‐axis indicates the number of pre‐outbreak codon changes from the ancestor to SARS‐CoV‐2 and RaTG13. The *y*‐axis indicates the number of codon changes that occurred during the pandemic. A blue dot indicates that a nonpreferred codon was replaced by a preferred codon (optimization). A red dot indicates that a preferred codon was replaced by an unpreferred codon (deoptimization). The red x shapes indicate the codon deoptimization caused by C>U mutations. The ten synonymous codon changes that occurred more frequently during the pandemic than in the preoutbreak history (Fisher's exact test, *P*‐adj < 0.05) are labeled, and the associated dots or x shapes are larger.

To investigate the possible impact of C>U synonymous mutations on CAI, we computed the *w_ij_* parameter to measure the relative usage of a codon (*j*) among all the codons that encode a specific amino acid (*i*). The parameter *w_ij_* was calculated based on the occurrences of codons in the coding sequences and the gene expression profiles in 54 human tissues (Materials and Methods). Thus, *w_ij_* = 1 means codon *j* is the most used in the transcriptomes among all the codons encoding amino acid *i*. Notably, all the synonymous changes generated by C>U deoptimize SARS‐CoV‐2 codons (that is, reduce the *w* value). The top 30 most abundant synonymous codon changes accounted for 90.7% of all the synonymous codon changes of a single nucleotide in the analyzed SARS‐CoV‐2 genomes, and 16 (53.3%) of such codon changes were caused by C>U mutations (**Figure** **2**a). Consequently, the C>U substitutional bias considerably contributed to the decreased CAI value in SARS‐CoV‐2.

Nucleotide substitutional bias was observed in the evolution of coronaviruses closely related to SARS‐CoV‐2 before the COVID‐19 pandemic.^[^ ^10^ ^]^ To examine whether the relative frequencies of nucleotide substitutional types differ between the pre‐outbreak history of SARS‐CoV‐2 in animals and its continuing evolution in humans, we inferred the ancestral state of each codon in the most recent common ancestor of RaTG13 and SARS‐CoV‐2, as previously described,^[^ ^20^ ^]^ and determined the frequencies of synonymous codon changes from the ancestral state to RaTG13 or SARS‐CoV‐2 (to reduce the stochastic noise, we pooled the synonymous changes in the two branches). As shown in Figure 2b, the occurrence of synonymous codon changes was significantly positively correlated with the pre‐outbreak evolution history in animals and the post‐outbreak evolution of SARS‐CoV‐2 in humans (Spearman's *ρ* = 0.73, *p* < 10^−10^). We found at least ten synonymous codon changes that were significantly enriched in SARS‐CoV‐2 compared with pre‐outbreak SARS‐CoV‐2‐like viruses; notably, 6 of them are C>U changes, which tend to deoptimize codon usage (Figure 2b). Among the four remaining types of synonymous codon changes, one is a G>A substitution, which might be caused by C>U editing in the antisense sequence, and one is an A>G change, which is putatively caused by A‐to‐I editing mediated by ADAR enzymes (Figure 2b). Considering that the genome sequence of BANAL‐20‐52 recently isolated in Laos is most similar to SARS‐CoV‐2,^[^ ^21^ ^]^ we also obtained the synonymous codon changes from the most recent common ancestor of BANAL‐20‐52 and SARS‐CoV‐2 to evaluate the pre‐outbreak evolution history (Figure S2, Supporting Information). Excessive synonymous mutations due to C>U substitution were still observed in the SARS‐CoV‐2 genomes, although two C>U changes (GCC>GCU and AUC>AUU) were not enriched in the SARS‐CoV‐2 genomes (Figure S2, Supporting Information). Overall, these results suggest that excessive C>U changes have accumulated in the SARS‐CoV‐2 genomes after the virus changed hosts from animals to humans, presumably because of the different host environments.

### 2.3 The High‐Frequency Synonymous Substitutions in SARS‐CoV‐2 are Dominated by C>U Changes

**Figure 3:**
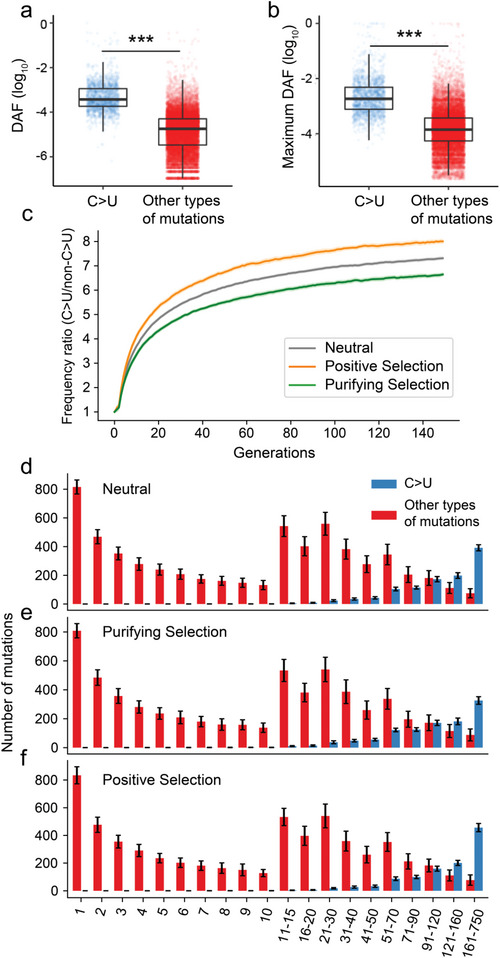
Simulation of viral sequences under biased mutation and different selections. a) The C>U synonymous mutations had significantly higher DAFs than the other types of synonymous mutations. b) The C>U synonymous changes had a significantly higher maximum DAF than the other types of synonymous changes. c) The DAF ratio of C>U mutations compared to non‐C>U mutations in the viral population under neutral (grey), positive selection (purple), and purifying selection (black) conditions during the simulation process. For each simulation replicate, the mean DAF values for C>U and non‐C>U mutations were computed independently at each generation. Subsequently, the ratio of the DAF value for C>U mutations relative to non‐C>U mutations at each generation was determined. The median and the 95% quantiles of the ratio were calculated based on 100 repetitions. d–f) The allele frequency spectrum of C>U and other types of mutations in the 150th generation under neutral or selective conditions. Mutations were grouped according to their observed number in the population, and the number of mutations in each group was tallied. The mean number of mutations in each group across 100 independent simulations was calculated and graphed, with error bars indicating standard deviation.

To explore the evolutionary forces acting on synonymous C>U changes, we first calculated the derived allele frequency (DAF) of the synonymous changes in all the SARS‐CoV‐2 genomes. In total, 19004 distinct synonymous changes were observed in 10214 sites, with 5473 sites having one type of synonymous change, 692 sites showing two types of synonymous changes, and 4049 sites exhibiting three types of synonymous changes, indicating that many of these synonymous changes are the result of independent recurrent mutations. The DAF values ranged from 1.09×10^−7^ to 0.994, with a median value of 2.22×10^−5^ and 2.5% and 97.5% quantile values of 2.18×10^−7^ and 1.40×10^−3^, respectively. Notably, the C>U synonymous changes had significantly higher DAFs than other types of synonymous changes (*p* < 10^−16^), and the median DAF of the former was more than 20 times that of the latter (3.75×10^−4^ *vs*. 1.80×10^−5^, **Figure** **3**a). We also utilized a second method to minimize the potential bias in estimating DAFs. Briefly, we binned the SARS‐CoV‐2 genomes collected every 14 days, calculated the DAFs of the synonymous changes in each bin, and then used the maximum DAF of a specific change to represent its DAF. Using this method, we obtained the same result, finding that the DAFs of the C>U synonymous changes were significantly higher than those of other types of synonymous changes (*p* < 10^−16^) and that the median DAF of the former was 13.1 times that of the latter (1.84×10^−3^ *vs*. 1.41×10^−4^, Figure 3b). These patterns persisted when we analyzed the Delta or Omicron variants separately (Figure S3, Supporting Information). These findings suggest that C>U synonymous substitutions, which typically deoptimize codons, have higher allele frequencies in SARS‐CoV‐2 populations than other types of synonymous substitutions.

Both mutational bias and natural selection may influence the frequency of C>U synonymous mutations; however, the precise role that natural selection plays in the accumulation of C>U synonymous mutations within SARS‐CoV‐2 genomes remains to be elucidated.^[^ ^13^, ^16^, ^18^, ^19^ ^]^ To explore whether mutational bias alone could account for the elevated DAFs of C>U synonymous mutations, we first conducted neutral sequence evolution simulations with a biased mutation model (Materials and Methods). In brief, our simulation considered the mutational bias based on the proportion of synonymous substitutions accumulated in the SARS‐CoV‐2 populations (Table S1, Supporting Information). In the simulations, neither natural selection nor recombination was considered, and the whole sequence evolution process was replicated 100 times. As shown in Figure S4a (Supporting Information), although the mean DAF increased for both C>U and other types of mutations as the number of generations increased, the mean DAF tended to be higher for C>U mutations, presumably resulting from independent recurrent mutations at the same sites.

To examine how natural selection influences the frequency of C>U mutations in addition to biased mutations, we further incorporated positive or purifying selection into the simulations. Specifically, in the purifying selection model, we retained the parameter settings of the neutral model and assumed a C>U mutation to be 10% less likely to be transmitted to the subsequent generation than other mutation types (Figure S4b, Supporting Information). In the positive selection model, we assumed that a C>U mutation was 10% more likely to be transmitted to the next generation than other mutation types (Figure S4c, Supporting Information). The results demonstrated that when C>U mutations were subjected to purifying selection, the ratio of the mean allele frequency of C>U mutations to that of other mutation types was considerably lower in each generation compared to the ratio under the neutral model. Conversely, when subjected to positive selection, the ratio of the mean allele frequency of C>U mutations to that of other mutation types was considerably higher in each generation compared to the ratio under the neutral model (Figure 3c). Based on the mutation spectra derived from these simulations, we conclude that C>U mutations tend to be enriched in higher frequency categories even under the neutral model (Figure 3d). This pattern persists even when C>U mutations are subject to purifying selection (Figure 3e). Moreover, the enrichment of C>U mutations in higher frequency categories becomes more pronounced when these mutations experience positive selection (Figure 3f).

Nevertheless, the DAF did not differ by more than ten times between these two categories of mutations in the simulations (Figure 3c; Figure S4, Supporting Information), in contrast to what was observed in actual SARS‐CoV‐2 populations. For example, at the 150th generation in our neutral simulations, the mean DAF was 14.3% for C>U mutations, while the corresponding number for other types of mutations was 2.0%. Thus, although C>U mutation bias could lead to high frequencies for C>U mutations, natural selection might need to be invoked to account for the observed difference in DAF between C>U and the other types of synonymous changes. It should be emphasized that the DAFs of synonymous alterations are influenced by confounding factors such as sampling bias in SARS‐CoV‐2 genome sequencing, the phylogeny of the viral strains, and the recurrent displacement of preexisting SARS‐CoV‐2 lineages by novel ones throughout the COVID‐19 pandemic. Consequently, it is challenging to estimate the distribution of synonymous mutational effects in the absence of strictly neutrally evolving sites. However, it is not unexpected that a substantial number of C>U and other classes of synonymous mutations are subjected to purifying selection, given the remarkably low DAF values of these mutations. On the other hand, positive selection that favors a C>U mutation might further elevate its frequency (Figure 3c; Figure S4, Supporting Information). We found 29 synonymous mutations that have DAF values greater than the mean + 3×s.d. (0.0343) of the DAF values of all sites (Table S2, Supporting Information), with C>U significantly overrepresented among these changes (18/1670 versus 11/17334; *P* = 1.4×10^−12^, Fisher's exact test). It is likely that these high‐frequency synonymous changes have been driven by positive selection, which is consistent with the results of our simulations, indicating that the frequencies of C>U mutations become elevated when subjected to positive selection (Figure 3f).

### 2.4 Experimental Verification of Translational Regulation of Synonymous Changes

**Figure 4:**
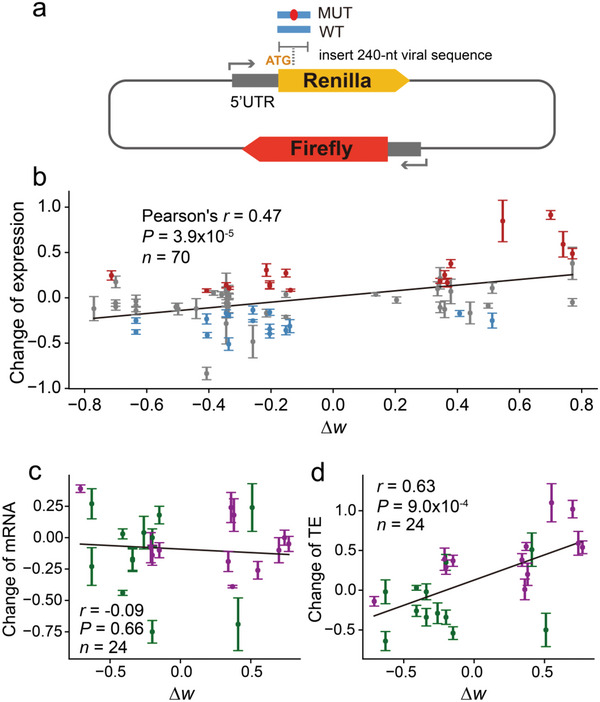
Effects of synonymous viral mutations on protein synthesis rate. a) The design of the dual‐luciferase reporter assays. By inserting a 240‐nt or 243‐nt viral CDS (centered on the mutation) after the Renilla start codon, two reporter plasmids (WT and MUT) were created. The function of the mutation was inferred by comparing the protein expression levels of MUT and WT. b) The correlations between protein expression changes and codon usage change by synonymous mutations. The *y*‐axis is the fold change (log_2_) of protein production of the mutant allele relative to that in the wild‐type allele. The *x*‐axis represents the change in *w* after codon substitution between a total of 70 pairs of WTS and MUT. The mean and standard errors of the change in protein expression level are presented for each mutation. Points are marked in red or blue when the relative intensity of the mutant allele is significantly higher or lower than that of the wild‐type allele (one‐sided t‐test, *p* < 0.05) and in gray if there is no significant difference. c) The relationship between changes in mRNA levels following a mutation and alterations in codon usage. The fold change (log_2_) of mRNA levels post‐mutation to pre‐mutation is plotted on the y‐axis. d) Translation efficiency changes resulting from alterations in protein and mRNA levels after a mutation (log_2_) were calculated. The top 12 mutations with the greatest increase in fluorescence level after the mutation are denoted by magenta dots, while the top 12 mutations with the greatest decrease in fluorescence level are denoted by green dots. Standard error bars are included, and the black solid line represents the fitted mean for each mutation.

Synonymous mutations potentially influence protein translation efficiency by optimizing or deoptimizing codons. To determine whether synonymous mutations in SARS‐CoV‐2 affect protein expression, we chose 70 synonymous mutations, including 8 with elevated DAF values (Table S2, Supporting Information) and 62 with relatively high frequencies, and used a dual‐luciferase reporter assay (psiCHECK‐2 vector) to analyze their effects (Table S3, Supporting Information). Briefly, for each synonymous mutation, we constructed two reporter plasmids with the wild‐type (WT) or mutant (MUT) allele and the flanking sequences (either 117 or 120 nucleotides on each flank of the focal codon, resulting in a fragment encoding 80 or 81 amino acids) of SARS‐CoV‐2 immediately after the start codon of the Renilla luciferase (**Figure** **4**a). We hypothesized that the synonymous mutation might affect local ribosomal elongation during protein synthesis; therefore, selecting 237 (or 240) nucleotides surrounding the focal SNP‐containing codon might adequately capture codon usage impact on elongation while reducing the effects caused by mRNA secondary structure. The construction of the plasmid consists of two main steps: 1) linearization of the backbone DNA and 2) synthesis of the insert fragment. By designing primers upstream and downstream of the insertion site for reverse PCR, we obtained the entire linearized plasmid backbone. We designed primers by segmentation and successfully constructed the insert fragment (including WT and MUT) sequences by overlapping PCR. Finally, we assembled the insert and linearized backbone by Gibson assembly and successfully obtained WT and MUT vectors (Materials and Methods). The expression of Renilla in psiCHECK‐2 was altered if the mutation affected protein synthesis. We measured the fluorescence intensities of Renilla and firefly luciferases. By comparing the Renilla expression levels between the WT and MUT vectors, using the firefly levels for normalization, we calculated the effect of the occurrence of mutations on protein expression.

Among the 70 synonymous mutations we detected, 48 resulted in codon usage optimization, while the remaining 22 led to codon usage deoptimization. We discovered a significant positive correlation between the change in protein expression level and the change in *w* (Δ*w*) for a synonymous mutation (Pearson's *r* = 0.47, *P* = 3.9×10^−5^; Figure 4b), supporting the notion that codon optimization tends to increase viral protein synthesis and codon deoptimization tends to decrease viral protein synthesis. Of note, although protein expression levels and Δ*w* exhibited a significant positive correlation, not all codon‐optimizing mutations with Δ*w* > 0 led to an increase in protein yield. Conversely, not all negative Δ*w* values resulted in decreased protein production. The effect of a particular synonymous mutation on protein expression level may be influenced by other factors, such as the surrounding sequence context of the codon, the mRNA secondary structure, and the CDS position in which the mutation resides.

We found that 17 of the 70 synonymous mutations significantly increased protein translation (one‐sided t‐test, *p* < 0.05; red points in Figure 4b) and 16 significantly decreased protein translation (one‐sided t‐test, *p* < 0.05; blue points in Figure 4b). Notably, we previously discovered that, based on two tightly linked mutations (the C8782U synonymous mutation and the U28144C nonsynonymous mutation), the SARS‐CoV‐2 genomes could be divided into an S lineage (U8782 and C28144) and L lineage (C8782 and U28144), with the S lineage as the ancestral and the L lineage as derived.^[^ ^20^ ^]^ According to our luciferase reporter assay, the derived C8782 allele found in the reference genome (L lineage) has lower translational efficiency than the ancestral U8782 allele found in the S lineage. Furthermore, the C15324U synonymous mutation, which had a high frequency in the B.1 variant common in Basel, Switzerland, in early 2020, decreases protein expression levels. In addition, the C29362U synonymous mutation, which had a high frequency in the Epsilon variant, increases protein expression. Overall, these results support the notion that synonymous mutations in SARS‐CoV‐2 might affect viral fitness or transmission by modulating viral protein translation.

In this study, the effect of a single synonymous mutation on protein expression was measured by analyzing pair of constructs with either WT or MUT viral fragments fusing with a fluorescent protein reporter. The effect of a single nucleotide change on the structure and stability of the mRNA might be present, albeit limited. We postulate that the increase in protein yield is primarily achieved through the enhancement of the local elongation rate of ribosomes. To test this hypothesis, we selected the 24 synonymous mutations (out of the aforementioned 70 mutations assessed via luciferase assays, as depicted in Figure 4b) that showed the most substantial effects on protein expression levels, including 12 upregulating and 12 downregulating mutations (Table S4, Supporting Information). These constructs were subjected to quantitative PCR (qPCR) analysis to determine mRNA levels, and the corresponding translation efficiencies were computed as the ratio of protein level to mRNA level. As shown in Figure 4c, no significant correlation was observed between Δ*w* and the change in the mRNA expression levels of the reporter genes (Pearson's *r* = ‐0.09, *P* = 0.66). Nevertheless, a significant positive correlation was found between the change in translation efficiency (adjusting for changes in protein expression levels using changes in mRNA levels) and Δ*w* (*r* = 0.63, *p* = 9.0×10^−4^; Figure 4d). These results suggest that the synonymous mutations potentially modulate protein expression of SARS‐CoV‐2 by altering translation elongation rather than affecting mRNA stability. Moreover, we also determined whether the length of viral fragment insertion might influence the translation elongation rate of the reporter gene. Two sets (A20055G and C13168U) of 483‐nt (80 amino acids on each side of the mutated codon) virus fragments were fused with the reporter gene, and their expression levels of the reporter gene were evaluated. In accordance with the 243‐nt fragment, the 483‐nt viral fragment insertion exhibited a similar effect (Figure S5, Supporting Information), indicating that the 243‐nt fragment insertion may be sufficient to assess the impact of synonymous mutations on protein synthesis.

### 2.5 Optimizing Codons of the S Gene of SARS‐CoV‐2 for Vaccine Design

**Figure 5:**
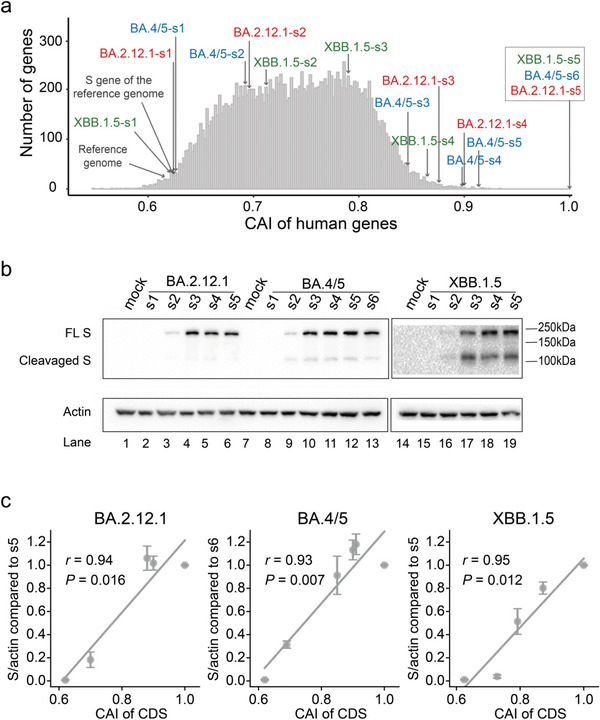
Experimental validation of codon optimization of the *S* gene. a) The distribution of CAI values of human genes and the partially or fully optimized codons for the S gene of SARS‐CoV‐2. For the S gene of BA.2.12.1, BA.4/5, and XBB.1.5 variants in the Omicron lineage, s1 represents the native viral CDS sequence, s2, s3, s4 and BA.4/5‐s5 represent the sequences with different CAI optimization levels, respectively, BA.2.12.1‐s5, BA.4/5‐s6, and XBB.1.5‐s5 represent the fully optimized CDS sequence that had a CAI value of 1. b) Western blotting analysis of S protein expression in HEK293T cells. The bands of full‐length and cleaved S protein are labeled. All the samples were probed using polyclonal rabbit anti‐SARS‐CoV‐2 S antibody (40591‐T62, Sino Biological) at a dilution of 1:2000. c) Correlation analysis between CAI value and S protein expression level. Experiments were repeated three times, error bars indicate stand error of the mean.

Codon selection is an essential consideration in vaccine design because codon usage affects the efficiency of protein production in vaccine development, thereby affecting antigen expression.^[^ ^22^ ^]^ The coronavirus Spike (S) protein binds the host receptor to allow viral entry into host cells and determines host ranges and tissue tropism. Moreover, the S protein is also the major target for neutralizing antibodies produced by host organisms. Given the importance of the S protein, many mRNA and protein subunit vaccines against SARS‐CoV‐2 have been designed to target this protein. However, the coding sequences of the *S* gene from the reference SARS‐CoV‐2 genome and the VOC/VOI genomes had low CAIs. For example, the CAI of the *S* gene in the reference genome was 0.624, and those of the BA.2.12.1 (BA.2.12.1‐s1), BA.4/BA.5 (BA.4/5‐s1) and XBB.1.5 (XBB.1.5‐s1) variants in the Omicron lineage were 0.624, 0.626, and 0.625, respectively. On the other hand, human genes had a median CAI value of 0.742, with 2.5th and 97.5th percentiles of 0.636 and 0.842, respectively (**Figure** **5**a). These comparisons highlighted the importance of optimizing the codon usage of the *S* gene for SARS‐CoV‐2 vaccine design in human or other mammalian cells.

To further evaluate the effect of codon usage on S protein expression, we developed an algorithm for codon optimization and constructed several plasmids encoding the S protein of the Omicron variant with mammalian codon usage optimized to varying degrees. We included S proteins of the BA.2.12.1, BA.4/5, and XBB.1.5 variants in our study. For each variant S protein, the encoding amino acid sequences from different constructs were identical, but their CAI values were different (Figure 5a). The levels of their expression were compared with those of constructs with the native sequence derived from the viral genome (BA.2.12.1‐s1 BA.4/5‐s1, and XBB.1.5‐s1) and the fully optimized sequence with a CAI value of 1 (BA.2.12.1‐s5, BA.4/5‐s6, and XBB.1.5‐s5) by Western blot analysis. For each S protein, we also included three randomly selected partially optimized sequences (s2, s3, s4, or s5) with CAI values ranging from 0.680 to 0.950 in the analyses.

As shown in Figure 5b, the original viral S protein coding sequence showed only a background level of S protein expression (BA.2.12.1‐s1 in lane 2, BA.4/5‐s1 in lane 8, and XBB.1.5‐s1 in lane 15). In contrast, the optimized sequences resulted in a high level of S protein expression for the BA.2.12.1 (s5 in lane 6), BA.4/5 (s6 in lane 13), and XBB.1.5 (s5 in lane 19) variants. Overall, the levels of S protein expression were strongly correlated with the CAIs (Figure 5c). Furthermore, Western blotting analysis revealed that our codon optimization for the S protein of the Omicron variants led to S protein expression levels that were comparable to those achieved with the codon optimization strategy employed in the coding sequence of the full‐length S protein of the Moderna mRNA‐1273 vaccine ^[^ ^23^ ^]^ (Figure S6, Supporting Information).

Consequently, these findings underscore the importance of codon usage optimization in mRNA and DNA vaccine design.

## 3 Discussion

Typically, viruses rely on the cellular machinery of their hosts to execute biological functions such as translation. Therefore, the viral codon usage patterns can impact viral protein production in host cells, ultimately affecting replication and transmission. However, the functional consequences of synonymous mutations in SARS‐CoV‐2 are understudied and poorly understood. In this study, we showed that the CAI of SARS‐CoV‐2 sequences declined over time but increased with the dominance of the Omicron variant. Notably, codon optimization and deoptimization during the evolution of SARS‐CoV‐2 genomes were predominantly driven by amino acid alterations, whereas synonymous mutations had minimal impact. We discovered a substantial preference for C>U synonymous changes in the SARS‐CoV‐2 genome. Our population genetics simulations suggest that mutational pressure, in conjunction with natural selection, may serve as the primary driving force behind this pattern. Through dual fluorescence experiments, we showed that codon optimization through synonymous mutations increases viral protein synthesis and that codon deoptimization decreases viral protein synthesis. Our findings suggest that the deoptimization effects of C>U synonymous mutations might be offset by other types of synonymous mutations that typically optimize codons, affecting CAI values of SARS‐CoV‐2 and resulting in a pattern where synonymous mutations contribute minimally to CAI changes throughout the COVID‐19 pandemic. We also provide experimental evidence that optimizing the codons of the *S* gene of SARS‐CoV‐2 variants enhances protein translation in human cells, which has important implications for vaccine design.

Our findings imply that synonymous mutations might potentially contribute to phenotypic disparities among distinct SARS‐CoV‐2 variants. Consequently, it is plausible that certain synonymous mutations might play a role in the emergence of novel circulating variants. For instance, the Delta variant harbors two specific synonymous mutations, C8986U (GAC>GAU) in *nonstructural protein 4* (*Nsp4*) and A11332G (GUA>GUG) in *Nsp6*. The Omicron variant possesses four unique synonymous mutations: C25000U (GAC>GAU) in the *S* gene, C25584U (ACC>ACU) in *ORF3a*, A27259C (AGG>CGG) in *ORF6*, and C27807U (CUA>UUA) in *ORF7b*. However, whether these synonymous mutations have any direct effect on SARS‐CoV‐2 pathogenesis and transmissibility warrants further investigation.

Host organisms have evolved multiple defense systems against viruses. APOBEC family enzymes, for example, introduce C>U mutations into viral RNA genomes. We found that C>U mutations are more prevalent in SARS‐CoV‐2 genomes in human populations than in related strains collected before the outbreak, presumably due to the host shift from nonhuman animals to humans. Notably, all the synonymous mutations generated by the C>U substitutions deoptimize the codons for translation in humans, and this may be a key mechanism that humans have evolved to defend against viruses. On the other hand, the large number of mutations in SARS‐CoV‐2 caused by RNA editing systems may supply viruses with an abundance of raw materials for adapting to human host conditions. Thus, RNA editing mechanisms also create a disadvantage that SARS‐CoV‐2 can exploit. It is plausible that SARS‐CoV‐2 exploits the host's antiviral system and deoptimizes codon usage to better adapt to the host's cells. To investigate whether such a hypothesis might apply to other coronaviruses, we analyzed complete genome sequences of four representative coronaviruses, including human coronavirus OC43 (HCoV‐OC43, *n* = 240), HCoV‐NL63 (*n* = 67), HCoV‐229E (*n* = 51), and HCoV‐HKU1 (*n* = 32) obtained from previous studies (Materials and Methods). Strikingly, the fractions of C>U and U>C changes were comparable and much higher than other types of mutations in all four coronaviruses, and this pattern remained consistent when analyzing all SNPs or only synonymous SNPs (Figure S7, Supporting Information). The enrichment of C>U mutations in these coronavirus strains might reflect editing by the APOBEC family enzymes, while the excessive U>C mutations might be caused by A‐to‐I RNA editing in the antisense strand during the replication of RNA genomes, as A‐to‐I editing frequently occurs in coronaviruses.^[^ ^10c^ ^]^ We noted a substantial rise in the proportion of C>U mutations accumulated in HCoV‐OC43 viral genomes over time. However, discerning patterns for HCoV‐NL63, HCoV‐229E, and HCoV‐HKU1 was challenging due to the limited number of available genome sequences (Figure S8, Supporting Information). Together, these observations lend additional support to the hypothesis that host organisms may exploit RNA editing as a defense mechanism against coronaviruses, consequently leaving signatures of biased nucleotide changes within viral RNA genomes during the evolutionary arms race processes.

Instead of evolving to utilize more human‐optimized codons, SARS‐CoV‐2 exhibited an overall lack of codon optimization. Although intuitively, one might expect higher translational efficiency to benefit the virus, it is possible that a high level of translation activity of viral mRNAs would cause a level of stress on the translational machinery of the host cells that does not ultimately benefit the virus. In accordance with this hypothesis, we found that the mRNA levels of SARS‐CoV‐2 tended to be much higher than those of mammalian genes by analyzing previously published mRNA‐Seq data. Specifically, we analyzed RNA‐seq data from Vero E6 cells infected with SARS‐CoV‐2 and harvested at 4, 6, 12, and 24 hours postinfection^[^ ^24^ ^]^ and RNA‐seq data from Vero cells (ATCC, CCL‐81) infected with SARS‐CoV‐2 and harvested at 24 h postinfection.^[^ ^25^ ^]^ In both datasets, we compared the expression levels (transcripts per kilobase of exon model per million mapped reads, TPM) of the top 3000 most abundantly expressed cellular genes to those of the SARS‐CoV‐2 genes. Notably, the TPM values were significantly higher for SARS‐CoV‐2 genes than for cellular genes. In the first dataset,^[^ ^24^ ^]^ the median TPM was 61.3, 6444, 35049, and 42061 for the SARS‐CoV‐2 genes at 4, 6, 12, and 24 h postinfection, while the median TPM was 68.4, 51.8, 2.42, and 0.2 for the top 3000 most abundantly expressed cellular genes at those time points, respectively (Figure S9, Supporting Information). Similarly, in the second dataset,^[^ ^25^ ^]^ the median TPM was 25209 for SARS‐CoV‐2 genes but only 1.136 for the top 3000 most abundantly expressed cellular genes, a ratio of 22191 (Figure S9, Supporting Information). We also retrieved 23 metagenomic datasets from the patients. In 20 of these samples, the RNA levels of SARS‐CoV‐2 genes were significantly higher than those of the top 3000 most abundantly expressed human genes after multiple‐test correction (Figure S10, Supporting Information). Collectively, these data suggest that SARS‐CoV‐2 mRNAs are highly abundant in host cells, even surpassing most host genes.

SARS‐CoV‐2 employs various strategies to impede host protein synthesis. For instance, Nsp1 could effectively suppress the global translation of host mRNA but not viral mRNAs due to unique features of the viral 5' untranslated region (UTR) in translation.^[^ ^26^ ^]^ Nsp16 of SARS‐CoV‐2 could bind to the mRNA recognition domains of U1/U2 RNA components of the spliceosome, leading to disruptions in global mRNA splicing and a reduction in the production of mature nascent RNA.^[^ ^26c^ ^]^ Additionally, ORF6 and Nsp1 have been found to obstruct the export of mRNA from the nucleus to the cytoplasm by binding to mRNA export factors Rae1 and NXF1, respectively, which ultimately inhibits the production of host proteins.^[^ ^27^ ^]^ Despite these strategies, according to the ribosome profiling studies of SARS‐CoV‐2‐infected cells, the translation efficiency of SARS‐CoV‐2 mRNA was not higher than that of cellular mRNAs overall and even declined over time.^[^ ^26d^ ^]^ Therefore, it is conceivable that elevated translation activity for viral mRNAs might not be essential for viral replication within host cells. The high abundance of viral transcripts, which leads to the high level of protein production of the SARS‐CoV‐2 genes,^[^ ^28^ ^]^ could facilitate virus translation to surpass host translation. Nevertheless, additional investigations are warranted to clarify the importance of codon usage bias in SARS‐CoV‐2 with respect to its translation, replication, and transmission.

Codon optimization is vital in vaccine development. The S protein of coronaviruses is the primary target of neutralizing antibodies of host organisms, and several mRNA and protein subunit vaccines of SARS‐CoV‐2 have been designed to target this protein. The different codon usage profiles in the SARS‐CoV‐2 genome relative to the human genome emphasizes the need to improve the codon usage for vaccine design against the SARS‐CoV‐2 S protein. We demonstrated that codon optimization of SARS‐CoV‐2 increases protein translation and substantially increases the levels of S protein expression in an experimental system. Consequently, our findings provide guidelines for codon usage optimization in mRNA and DNA vaccine formulation and are broadly applicable to the design of vaccines against other viruses and biopharmaceutical engineering.

## 4 Experimental Section

### SARS‐CoV‐2 Sequence Variants and Annotation

It was downloaded 9164789 high‐quality SARS‐CoV‐2 genomes from the Global Initiative on Sharing All Influenza Data (GISAID, https://www.gisaid.org; as of June 18, 2022). The SARS‐CoV‐2 genomes to the reference genome (NC_04 5512) using MAFFT v7.453 ^[^ ^29^ ^]^ with the default parameters and used SnpEff v5.0e ^[^ ^30^ ^]^ to annotate the synonymous and nonsynonymous substitutions was mapped.

The collection date and lineage annotation information for the SARS‐CoV‐2 genomes and calculated the proportions of VOCs and VOIs in 14‐day sliding windows with a one‐day step size was retrieved. The percentages of different nucleotide substitution types in the SARS‐CoV‐2 genomes collected in each time window was also calculated. For each synonymous substitution, the DAF of the synonymous changes in each time window was computed and used the maximum DAF of a specific change to represent its DAF. It was also calculated the DAF of each SNV across all the SARS‐CoV‐2 genomes.

### Ancestral Sequence Reconstruction

The coding sequences (CDSs) of SARS‐CoV‐2, RaTG13, BANAL‐20‐52, Pangolin GX‐P5L, and Pangolin GD MP789 from GenBank with the accession numbers NC_04 5512, MN996532, MZ937000, MT040335, and MT121216, respectively was downloaded. The protein sequences of each gene using MUSCLE v3.8.31 ^[^ ^31^ ^]^ and then reverse‐translated them into codon alignments using RevTrans was first aligned.^[^ ^32^ ^]^ It was concatenated all the aligned CDSs of each genome and reconstructed the phylogenetic tree based on the neighbor‐joining algorithm and the Jones‒Taylor‒Thornton (JTT) model using MEGA X.^[^ ^33^ ^]^ The CODEML program in the Phylogenetic Analysis by Maximum Likelihood (PAML) package ^[^ ^34^ ^]^ was used to reconstruct the sequences of the most recent common ancestors. The synonymous codon changes from the most recent common ancestor of SARS‐CoV‐2 and RaTG13 (or BANAL‐20‐52) to SARS‐CoV‐2 and RaTG13 (or BANAL‐20‐52) were defined as pre‐outbreak codon changes.

### Calculation of the CAI of SARS‐CoV‐2

The classic CAI was calculated based on the codon usage profile of the highly expressed genes.^[^ ^35^ ^]^ In this study, expression‐weighted CAI in humans based on the actual frequency of codon usage in the transcriptomes was calculated. The gene expression profiles of 54 human tissues from the Genotype–Tissue Expression (GTEx) database Version 8 (https://www.gtexportal.org/) was downloaded. The median expression value across tissues for each gene and used the median value as its expression level was calculated. The human genome sequence and annotations used in this study were downloaded from GENCODE (GRCh38.p13), and the longest transcripts of each gene were retained.

Combining the protein sequences and the mRNA expression levels, it was calculated the occurrences of codons in the transcriptome as follows:

(1) $$X_{ij}=\;\sum_{n\;=\;1}^{m}Codon_{ln}\times {\mathrm{TPM}}_{n}$$

where *X_ij_* represents the occurrence of the *j*th codon of the *i*th amino acid in the transcriptome, *Codon_ln_* represents the occurrence of the *l*th codon in the *n*th gene, TPM_*n*_ denotes the mRNA expression of the *n*th gene, and *m* denotes the number of genes included in the analysis.

Next, it was calculated the relative usage of each codon as follows:

(2) $$RSCU_{ij}=\frac{X_{ij}}{\frac{1}{n_{i}}\sum_{j=1}^{n_{i}}X_{ij}}\;$$

where *n_i_* indicates the number of codon types for the *i*th amino acid. Finally, it was calculated the *w_ij_* parameter as

(3) $$w_{ij}=\frac{RSCU_{ij}}{\max\left(RSCU_{i}\right)}\;$$

and further determined the CAI value of a sequence as the geometric mean of the *w_ij_* values for all the codons in that sequence. It was defined codons with *w_ij_* ≥ 0.9 as optimal and those with *w_ij_* < 0.9 as non‐optimal.

The coding sequences of SARS‐CoV‐2 (*ORF1ab*, *S*, *ORF3a*, *E*, *M*, *ORF6*, *ORF7a*, *ORF7b*, *ORF8*, *N*, and *ORF10*) to calculate the expression‐weighted CAI value was concatenated. To assess the effects of synonymous mutations on CAI, the sites with nonsynonymous mutations in the variant genomes with the corresponding sequences from the reference genome was replaced. To obtain the CAI values of the native *S* genes of three Omicron lineages (BA.4, BA.5, BA.2.12.1, and XBB.1.5), it was randomly selected one high‐quality sequence representing each lineage from the GISAID database (BA.4: EPI_ISL_13 802 998, BA.5: EPI_ISL_13 578 652, BA.2.12.1: EPI_ISL_13 524 674, and XBB.1.5: EPI_ISL_15 851 788). The sequences of *S* genes were identified using exonerate v2.4.0 ^[^ ^36^ ^]^

### Population Genetics Simulations

It was used forward simulations to analyze the distribution of derived allele frequencies of different mutational types. Briefly, the simulation begins with one randomly generated ancestral sequence with a length (*L*) of 6000 nucleotides, like the number of synonymous sites of SARS‐CoV‐2, and a nucleotide composition like those of the coding sequences of the reference genome of SARS‐CoV‐2 (A, 0.298582; U, 0.322160; C, 0.183222; G, 0.196036). In the 2nd generation, the ancestral sequence generated 5 random offspring viral sequences according to the mutation matrix presented in Table S1 (Supporting Information). This mutation matrix was obtained by calculation based on the proportion of mutations observed in the observed viruses. The relative proportions between the various bases were calculated by averaging the distribution of synonymous substitutions over a sliding window of 14 days in length. The lowest proportion, C>A, was assigned a value of 1 in 100 000, and the frequencies of other mutations were converted according to the proportions to be used in genetics simulations. We assumed different nucleotides to have different mutation rates based on the observations in SARS‐CoV‐2, with mutation rates of 2.31×10^−4^, 2.76×10^−4^, 1.28×10^−3^, and 2.58×10^−4^ per site per generation for A, U, C, and G, respectively. On average, the mutation rate used in this simulation was 4.44×10^−4^ per site per generation, which resulted in ≈2.66 mutations per offspring sequence. The mutation rate of each type of nucleotide to follow a Poisson distribution was assumed. In the third generation, each of the six viral sequences generates 5 random offspring viral sequences using the same parameter settings. The simulation continues to the next generation with the same parameter. When the population size exceeded 1000 (at the 5th generation and beyond), it was randomly selected 1000 parental and offspring sequences to continue with. The simulation was stopped after 150 generations. The whole simulation process was replicated 100 times, and the distribution of the derived allele frequency of each mutation type was summarized based on 100 replicates of the simulation. In addition to neutral evolution, it was incorporated positive or purifying selection into the simulations while maintaining the parameter settings of the neutral model. Briefly, in the purifying selection model, a C>U mutation to be 10% less likely to be transmitted to the subsequent generation compared to other mutation types was assumed. Conversely, in the positive selection model, it was assumed that a C>U mutation was 10% more likely transmitted to the next generation than other mutation types.

### Dual‐Luciferase Reporter Assay

A dual‐luciferase assay (psiCHECK‐2 vector, Promega) was performed to analyze the effects of the viral mutation on protein synthesis. The primers psi_backbone_F and psi_backbone_R were used to generate PCR products (of the vector backbone) with complementary overhangs. Following gel extraction, template plasmids were digested using *Dpn* I (NEB). The wild‐type and mutated viral sequences were constructed using overlap PCR and assembled with the backbone with the NEBuilder HiFi DNA Assembly Cloning Kit (E5520S, NEB). All primers used in this study were given in Table S3 (Supporting Information) and synthesized at RuiBiotech Company (Beijing, China). To construct viral fragments with homologous overhangs, we utilized WT‐1, WT‐2, WT‐3 (or MUT‐3), and WT‐4 primers in an overlap PCR strategy. As depicted in Figure S11 (Supporting Information), the red or green portions on the WT‐1 and WT‐4 primers represent 20nt‐long homologous overhangs used for connecting to the plasmid backbone. WT‐1 is ≈75 nt, while WT‐2, WT‐3, MUT‐3, and WT‐4 were ≈87 nt. The specific length of the primers may vary slightly when constructing different synonymous mutation vectors. The viral segment synthesis did not use a template sequence but was generated directly through two stages of PCR. The first stage involved amplification using the WT‐2 and WT‐3 primer pair, which contain ≈17 nt of complementary homologous sequence at their 3' ends. The second stage involved the addition of the WT‐1 and WT‐4 primers to the product of the first stage, with these primers containing ≈17 nt of homologous sequence with WT‐2 and WT‐3, respectively. The first stage involved 5 PCR cycles, while the second stage involved 25 PCR cycles. The final product is a wild‐type viral sequence fragment with homologous arms on both sides. MUT‐3 and WT‐3 differ by a single nucleotide, which can be used to construct a mutant viral sequence fragment following the same process as for the wild‐type. The complete viral fragment by gel recovery was obtained. Subsequently, it was employed Gibson assembly to ligate the products of the two processes. Finally, the resulting relevant plasmids by Sanger sequencing to ensure the absence of errors before proceeding to the subsequent functional experimental validation was verified.

HEK293FT cells were purchased from the Cell Bank of the Chinese Academy of Sciences and were maintained in Dulbecco's modified Eagle's medium (DMEM, Gibco) with 10% FBS (Gibco) and 1% penicillin‒streptomycin at 37 °C in a humid incubator with a 5% CO_2_ atmosphere. Plasmids were extracted using the QIAGEN Miniprep kit (27 106, QIAGEN) according to the manufacturer's instructions. The constructed vectors were into HEK293FT cells with the Lipofectamine 3000 Transfection Reagent (L3000015, Thermo Fisher Scientific) was transfected. Cells were cultivated for 32 h after transfection, and then a dual‐luciferase reporter assay system (Promega) was used to detect the Renilla levels of wild‐type and mutant plasmids and normalized to firefly luciferase as an internal control (five biological replicates per plasmid). Raw data was recorded in Table S3 (Supporting Information).

### Quantify the Impact of Mutations on mRNA Levels and Translation Efficiency

The cell culture and transfection procedures were like those used in the Dual‐Luciferase reporter assay described earlier. At 32 h post‐transfection, cells were lysed using TRIzol (Invitrogen), and total RNA was extracted according to the instructions provided by the manual. The extracted RNA was reverse‐transcribed with random primers using cDNA synthesis kit (6210, Takara), and qPCR was performed using PowerUp SYBR Green Mix (A25742, Applied Biosystems). Each plasmid was analyzed in triplicate (Table S4, Supporting Information). The log_2_ fold change in translation efficiency was determined by subtracting the log_2_ fold change in mRNA levels from the log_2_ fold change in fluorescence (protein) levels.

### Optimization of the Coding Sequence of the *S* Gene

To compare the impact of different optimization levels on protein expression, it was randomly introduced non‐optimal codons into the coding region without regional preference and at various proportions, obtaining sequences with CAI values ranging from 0.680 to 0.950 for experimental validation. For the encoding sequence with a CAI of 1, the most frequently used codons summarized in different human tissues to encode the amino acid sequence was employed. A specific optimization example was provided and shared on GitHub (https://github.com/Xinkai‐Wu/CodonOptimizationNotebook).

### Western Blot Analysis of S Protein Expression

The coding sequences of the S protein for BA.2.12.1, BA.4/5, or XBB.1.5 variants with various CAIs were synthesized by GeneScript (Nanjing, China) and cloned into the pcDNA 3.1 plasmid between the *Hind* III and *Xba* I sites. HEK293T cells were obtained from ATCC and maintained in DMEM with 10% FBS and 1% penicillin‐streptomycin at 37 °C and 5% CO_2_. Approximately 2×10^6^ HEK293T cells were transfected with 2 µg of plasmid encoding S proteins using polyetherimide (PEI; Sigma). After 16 h of incubation, the cells were fed a fresh medium. The next day, the cells were lysed with cell lysate buffer (20 mmol/L Tris‐HCl pH 7.5, 150 mmol/L NaCl, 1 mmol/L EDTA, 0.1% sodium dodecyl sulfate (SDS), 1% NP40, 1× protease inhibitor cocktail (Bimake, Houston, USA)) and centrifuged at 12000 × g for 10 min to remove nuclei. The cell lysates were boiled and separated by 10% SDS‐polyacrylamide gel electrophoresis (PAGE). After being transferred to nitrocellulose filter membranes (GVS, Sanford, USA), the samples were probed using polyclonal rabbit anti‐SARS‐CoV‐2 S antibody (40591‐T62, Sino Biological) at a dilution of 1:2000, or the polyclonal antibody against the S2 subunit of SARS‐CoV‐2 spike protein (40590‐T62, Sino Biological).

### The Proportion of Mutations Accumulated in Other Coronavirus Species

It was downloaded 241 human coronavirus OC43 (HCoV‐OC43), 68 HCoV‐NL63, 52 HCoV‐229E, and 33 HCoV‐HKU1 complete genome sequences from the NCBI virus database (https://www.ncbi.nlm.nih.gov/labs/virus/vssi/#/; as of February 1, 2023; Table S5, Supporting Information). Each sequence was aligned to the genome sequence with the earliest collection date (HCoV‐OC43: KF530060 collected on January 8, 1985; HCoV‐NL63: KF530110 collected on August 16, 1983; HCoV‐229E: KF514432 collected on February 22, 1993; HCoV‐HKU1: HM034837 collected on March 4, 2005) using MAFFT v7.453.^[^ ^29^ ^]^ Point mutations were obtained using SNP‐sites v2.5.1 ^[^ ^37^ ^]^ and subsequently annotated with SNPeff v5.0e.^[^ ^30^ ^]^ The proportion of each of the twelve single‐nucleotide substitution types for each viral genome sequence was calculated.

### Quantification of Viral and Cellular Gene Expression

It was retrieved RNA‐seq data from Vero E6 cells that were infected with SARS‐CoV‐2 at a multiplicity of infection (MOI) of 0.1 and harvested at 4, 6, 12, and 24 h postinfection ^[^ ^24^ ^]^ and RNA‐seq data from Vero cells (ATCC, CCL‐81) infected with SARS‐CoV‐2 at an MOI of 0.05 and harvested at 24 h postinfection.^[^ ^25^ ^]^ The gene expression of SARS‐CoV‐2 and the hosts in the Kallisto program was calculated,^[^ ^38^ ^]^ using the longest transcript of *Chlorocebus sabaeus* (ChlSab1.1) and the CDS of SARS‐CoV‐2 (NC_04 5512) as the reference transcriptomes. It was also retrieved 36 patient metagenomic datasets from the NCBI Short Read Archive (SRA) and kept 23 libraries with gene expression information for all the SARS‐CoV‐2 genes. The expression levels of the SARS‐CoV‐2 and human genes were also calculated by the Kallisto program.^[^ ^38^ ^]^

### Data and Code Availability

The accession numbers of the SARS‐CoV‐2 genome sequences analyzed in this present study have been deposited into https://github.com/Xinkai‐Wu/CodonOptimizationNotebook. The data and codes in this study were available by contacting the corresponding authors upon reasonable request.

## Conflict of Interest

The authors declare no conflict of interest.

## Supporting information

Supporting Information

Supplemental Table 1

Supplemental Table 2

Supplemental Table 3

## Data Availability

The accession numbers of the SARS‐CoV‐2 genome sequences analyzed in this present study have been deposited into https://github.com/Xinkai‐Wu/CodonOptimizationNotebook. The data and codes in this study are available in the supporting information and by contacting the corresponding authors upon reasonable request.
