# Supplementary material for: Optimization and Deoptimization of Codons in SARS‐CoV‐2 and Related Implications for Vaccine Development
Source: Adv Sci (Weinh). 2023 Jun 2;10(23):2205445. doi: 10.1002/advs.202205445 (PMC10427376; doi:10.1002/advs.202205445)
Supplement: Supplementary file 1 — Supporting Information [file ADVS-10-2205445-s003.pdf]

ADVANCED SCIENCE
Open Access

Supporting Information

for *Adv. Sci.*, DOI 10.1002/advs.202205445

Optimization and Deoptimization of Codons in SARS-CoV-2 and Related Implications for Vaccine Development

Xinkai Wu, Ke-jia Shan, Fuwen Zan, Xiaolu Tang, Zhaohui Qian and Jian Lu**

Supplementary Tables and Figures

Table S1. The mutational matrix used for population genetics simulation

		**Ancestral allele**			
		**A**	**U**	**C**	**G**
**Derived allele**	**A**	0.999769	0.000012	0.000010	0.000180
	**U**	0.000015	0.999724	0.001259	0.000062
	**C**	0.000035	0.000243	0.998716	0.000016
	**G**	0.000181	0.000021	0.000015	0.999742

Table S2. The 29 synonymous mutations in SARS-CoV-2 genomes that are putatively under positive selection

Position	Reference	Alternative	Gene	Protein change	Ref Codon	Alt Codon	DAF
913	C	U	ORF1ab	S216S	UCC	UCU	0.11
2470	C	U	ORF1ab	A735A	GCC	GCU	0.09
3037	C	U	ORF1ab	F924F	UUC	UUU	0.99
4321	C	U	ORF1ab	A1352A	GCC	GCU	0.16
5386	U	G	ORF1ab	A1707A	GCU	GCG	0.19
5986	C	U	ORF1ab	F1907F	UUC	UUU	0.11
8986	C	U	ORF1ab	D2907D	GAC	GAU	0.37
9424	A	G	ORF1ab	V3053V	GUA	GUG	0.15
10198	C	U	ORF1ab	D3311D	GAC	GAU	0.15
10447	G	A	ORF1ab	R3394R	AGG	AGA	0.16
11332	A	G	ORF1ab	V3689V	GUA	GUG	0.37
12880	C	U	ORF1ab	I4205I	AUC	AUU	0.16
13195	U	C	ORF1ab	V4310V	GUU	GUC	0.19
14676	C	U	ORF1ab	P4804P	CCC	CCU	0.11
15240	C	U	ORF1ab	N4992N	AAC	AAU	0.21
15279	C	U	ORF1ab	H5005H	CAC	CAU	0.11
15714	C	U	ORF1ab	L5150L	CUC	CUU	0.16
16176	U	C	ORF1ab	T5304T	ACU	ACC	0.11
17040	U	C	ORF1ab	N5592N	AAU	AAC	0.05
20055	A	G	ORF1ab	E6597E	GAA	GAG	0.15
21595	C	U	S	V11V	GUC	GUU	0.04
22792	C	U	S	I410I	AUC	AUU	0.06
25000	C	U	S	D1146D	GAC	GAU	0.34
25584	C	U	ORF3a	T64T	ACC	ACU	0.35
26858	C	U	M	F112F	UUC	UUU	0.15
27259	A	C	ORF6	R20R	AGG	CGG	0.35
27384	U	C	ORF6	D61D	GAU	GAC	0.16
27807	C	U	ORF7b	L18L	CUA	UUA	0.35
28882	G	A	N	R203R	AGG	AGA	0.50

Table S3 is an Excel file that contains the primers used for the construction of dual-luciferase assays, and the raw and processed data for all constructions. The eight synonymous variants listed in Table S2 were highlighted in red.

Table S4 is an Excel file that contains the primers used for qPCR assays, as well as the raw CT values obtained from qPCR assays.

Table S5 is an Excel file that contains the sequence information of four human coronaviruses (OC43, NL63, 229E and HKU1) used in this study.

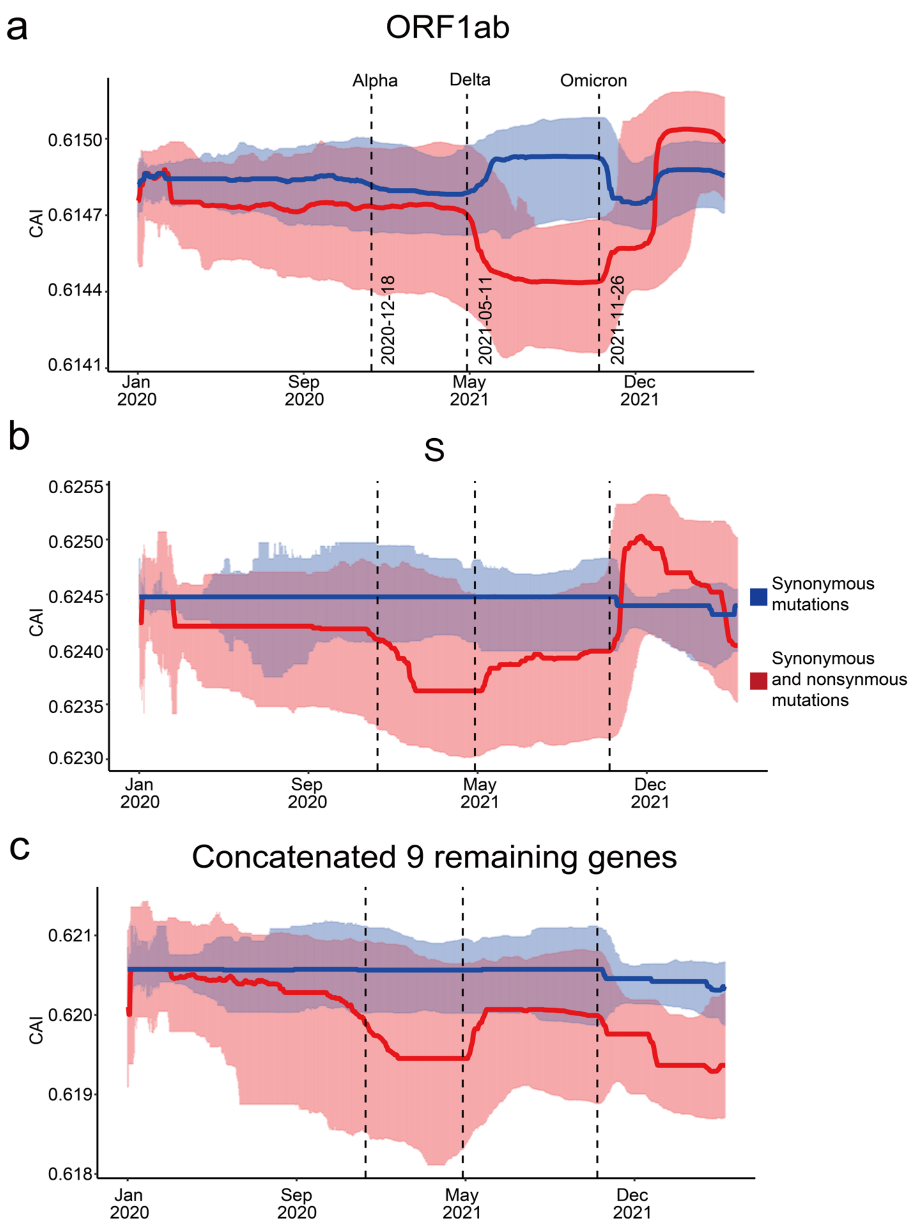

Figure S1. The CAI change of *ORF1ab* (a), *S* (b) and the concatenated nine remaining genes (c) of SARS-CoV-2 caused by synonymous and nonsynonymous (red) or synonymous mutations (blue) over time. The red and blue solid lines indicate the median of CAI, and the red and blue shadows indicate the 95% interval of CAI in a 14-days window with a one-day step. The black dash lines indicate the dates when World Health Organization defined the Alpha, Delta, and Omicron lineages as the variants of concern. Due to the nine remaining genes (*ORF3a*, *E*, *M*, *ORF6*, *ORF7a*, *ORF7b*, *ORF8*, *N*, and *ORF10*) are generally short and did not yield informative results, we concatenated the remaining nine genes, excluding the *ORF1ab* and *S* genes, for the CAI analysis.

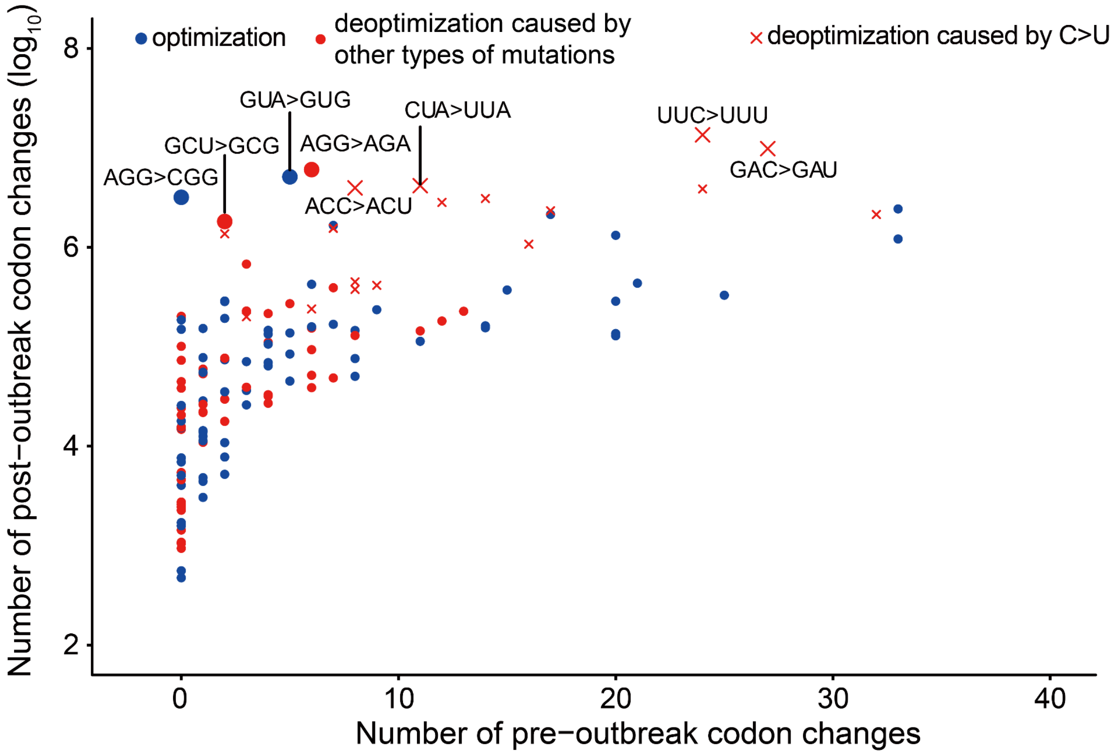

Figure S2. The post-outbreak (*y*-axis) over pre-outbreak (*x*-axis) synonymous codon change. The pre-outbreak codon changes were inferred based on the synonymous codon changes from the most recent common ancestor of BANAL-20-52 and SARS-CoV-2 to them in the pre-outbreak evolution history.

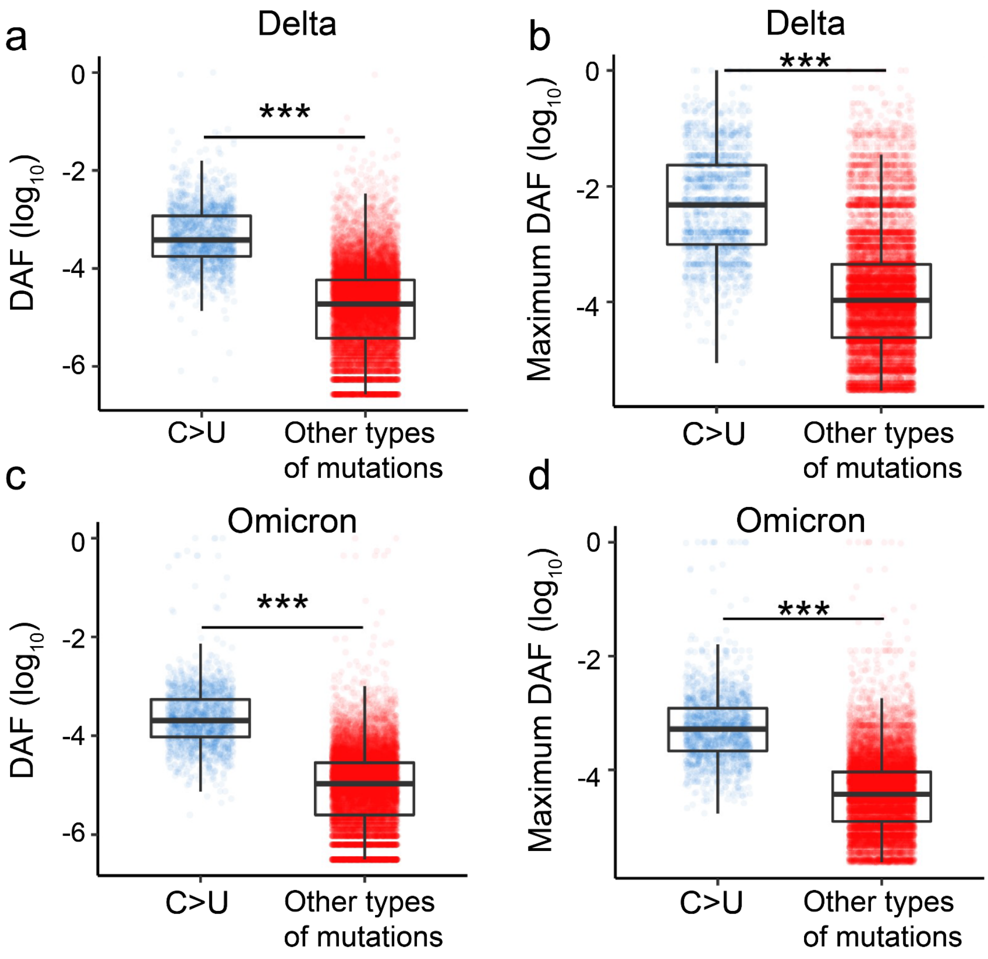

Figure S3. The DAFs of the C>U synonymous changes are significantly higher than those of the non-C>U synonymous changes in the Delta (a, b) or Omicron variants (c, d). (a, c) All the mutations were considered in the mutation frequency calculation. (b, d) The maximum DAF in a time window was used to represent the DAF of a synonymous mutation.

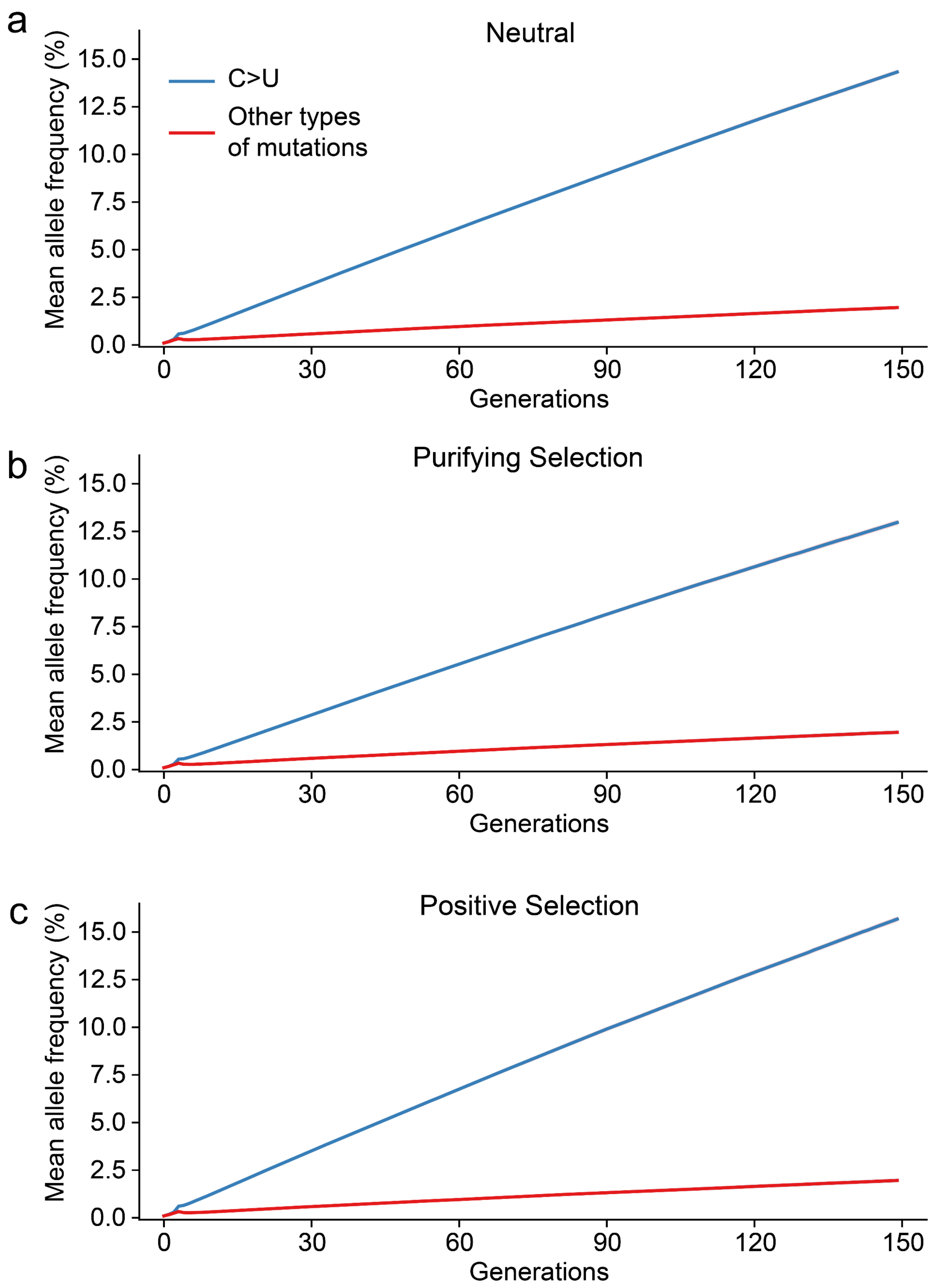

Figure S4. The DAF values of C>U and non-C>U mutations in the viral population under neutral (a), purifying selection (b), or positive selection (c) conditions during the simulation process. For each simulation replicate, the mean DAF values for C>U and non-C>U mutations were computed independently at each generation. The median and the 95% quantiles of the DAF values were calculated based on 100 repetitions.

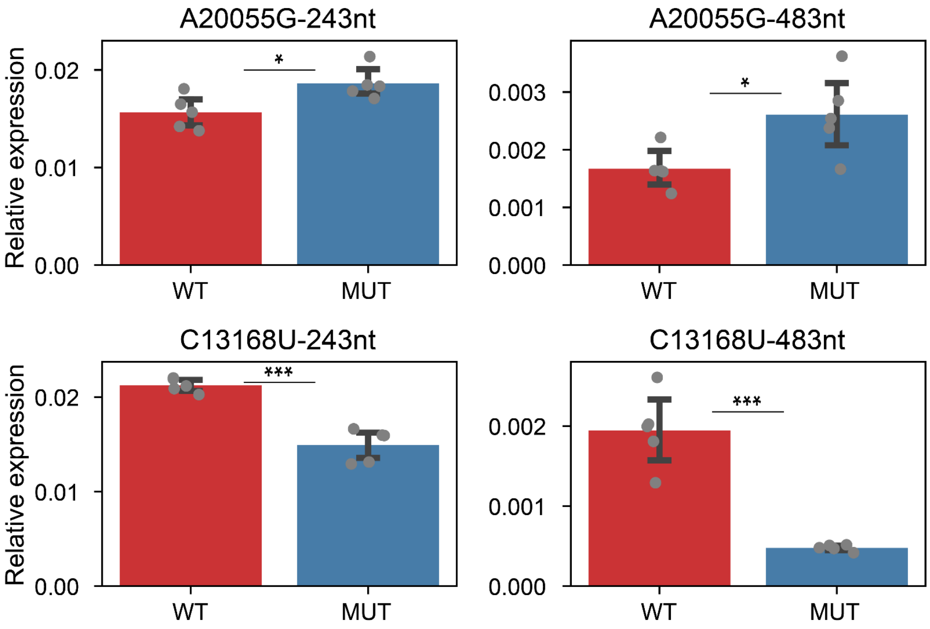

Figure S5. Assessment of the potential influence of viral fragment insertion length on the translation elongation rate of the reporter gene. For each of the two mutations (A20055G and C13168U), a 243-nt fragment insertion and a 483-nt viral fragment for the wild-type or mutant allele were fused with the reporter gene. The expression levels of the reporter gene were then evaluated for both fragment insertion lengths. The results demonstrated that the observed mutation effects were remarkably consistent across both length conditions. Each sample was repeated five times, and the standard errors were represented. *, $P \leq 0.05$, ***, $P < 0.001$.

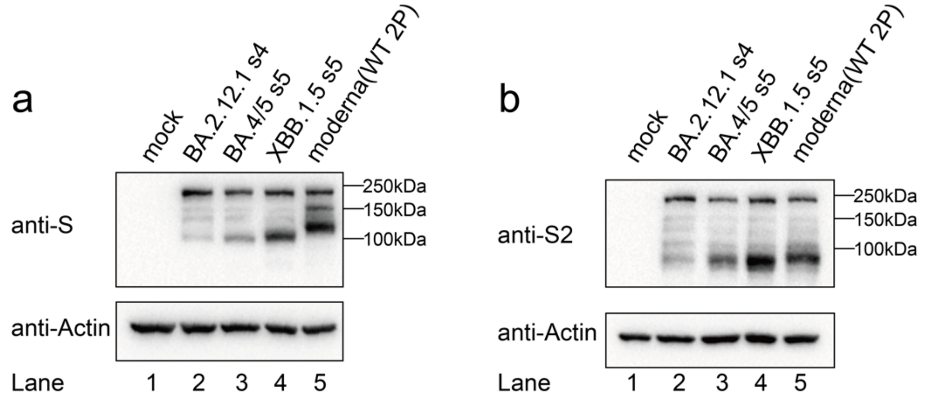

Figure S6. Western blot analysis for the protein abundance of the full-length S protein using our codon optimization strategy of the Omicron variants (BA.2.12.1, BA.4/5, and XBB.1.5) against the strategy employed in the coding sequence of the full-length S protein of the Moderna mRNA-1273 vaccine. (a) the samples were probed using polyclonal rabbit anti-SARS-CoV-2 S antibody (40591-T62, Sino Biological). (b) the samples were probed using the polyclonal antibody against the S2 subunit of SARS-CoV-2 spike protein (40590-T62, Sino Biological). WT 2P: the wild type of S protein with two proline substitutions in Moderna mRNA-1273 vaccine.

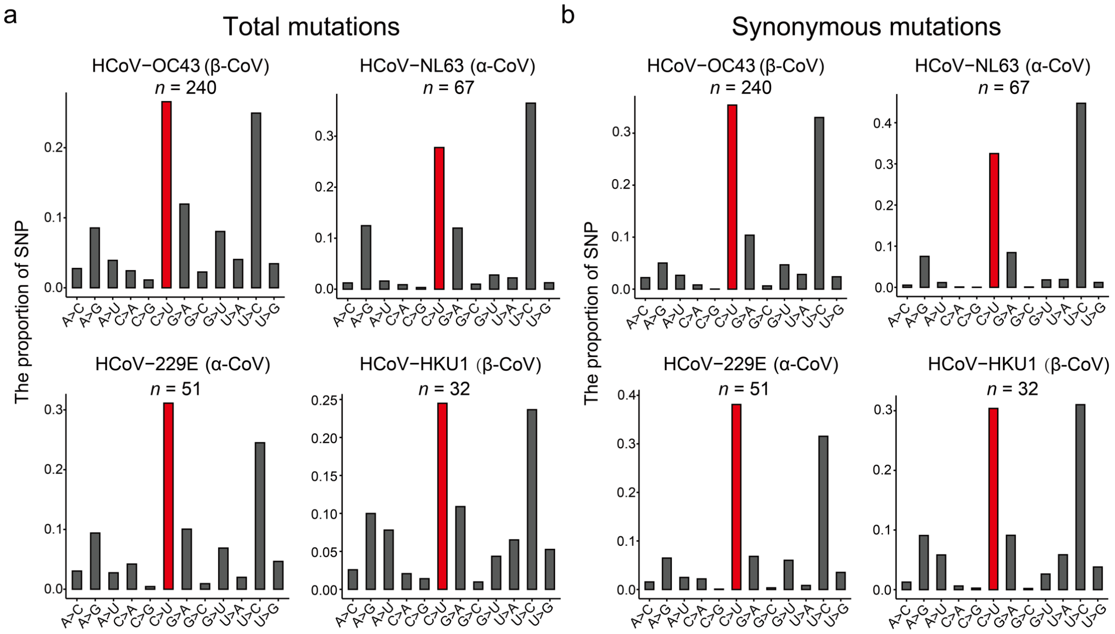

Figure S7. The proportions of each type of changes in the total (a) and synonymous (b) SNPs accumulated in the genomes of human coronavirus OC43 (HCoV-OC43), HCoV-NL63, HCoV-229E, and HCoV-HKU1. The genus name was indicated in the parentheses, and the corresponding sequence number under analysis was labeled below.

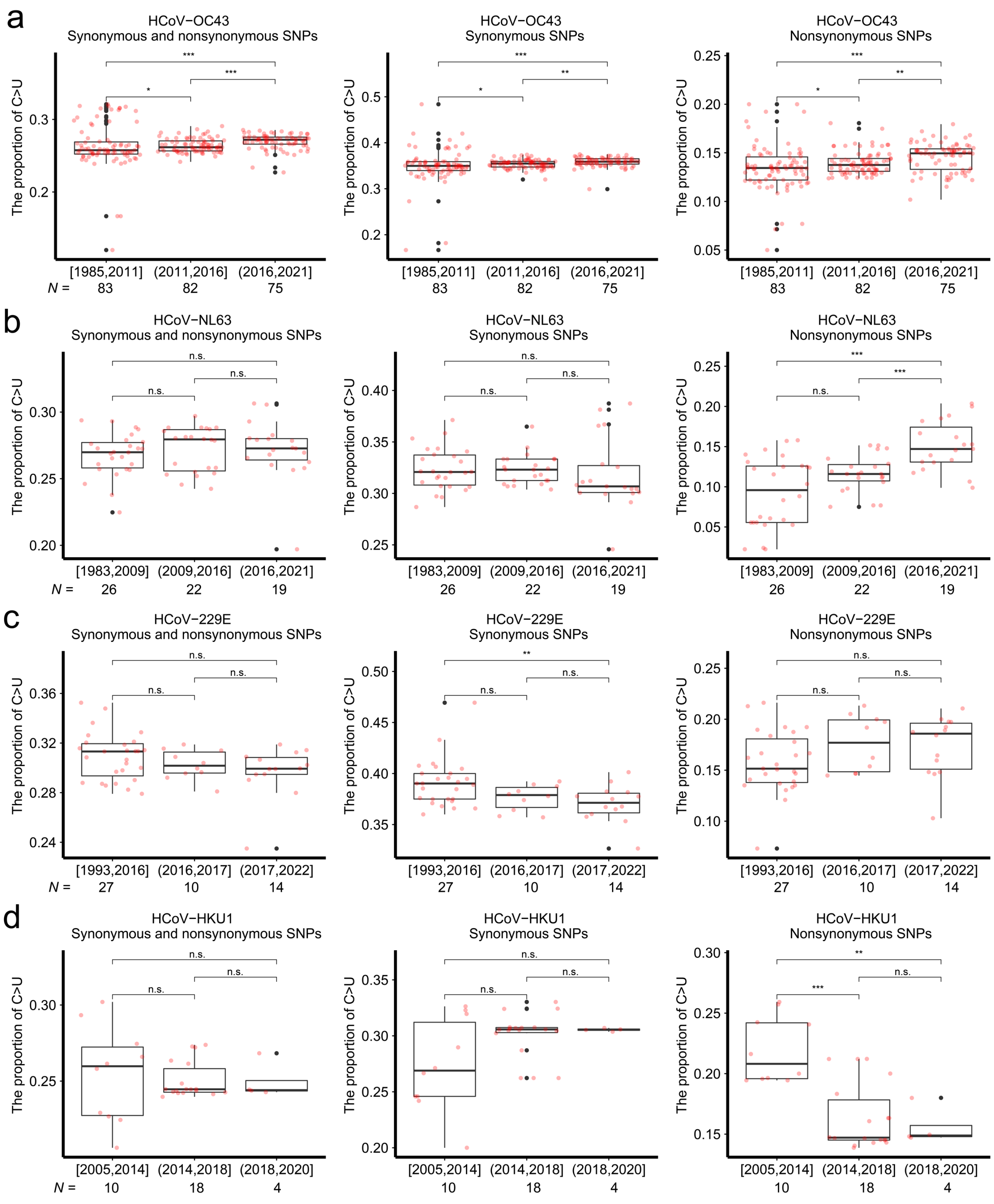

Figure S8. The proportions of C>U mutations accumulated in the genomes of human coronavirus OC43 (HCoV-OC43), HCoV-NL63, HCoV-229E, and HCoV-HKU1 over time. The sequence number was labeled below. n.s., $P > 0.05$; *, $P \leq 0.05$; **, $P < 0.01$; ***, $P < 0.001$.

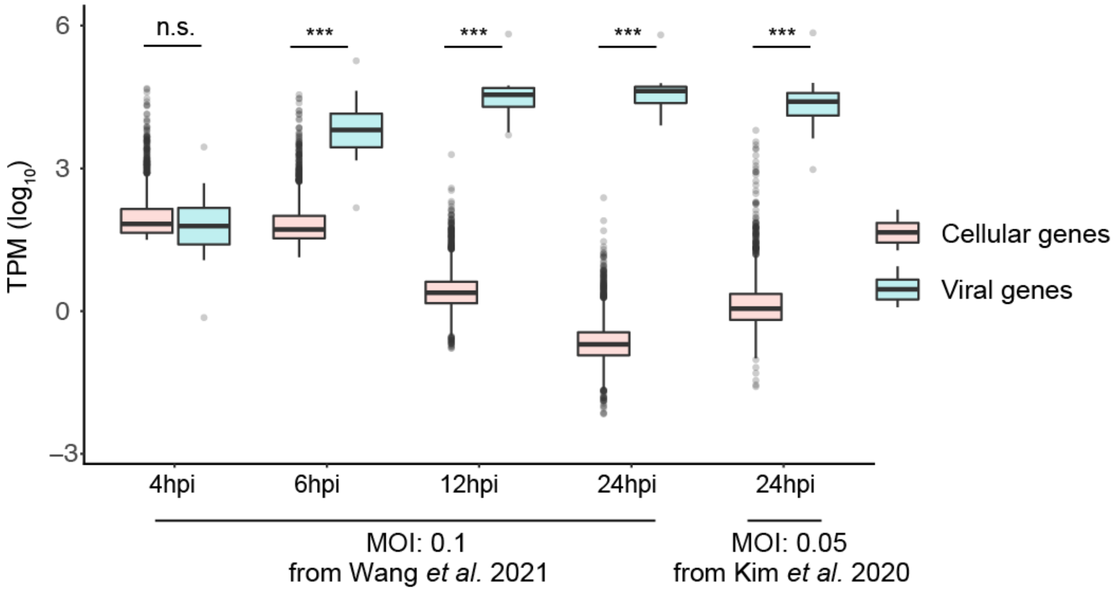

Figure S9. The gene expression levels of Vero cells infected with SARS-CoV-2 and harvested at different time points. For the Vero cells, only the top 3000 highly expressed genes were considered in the analysis. The initial multiplicity of infection (MOI) and the cultivation time (hours post-infection, hpi) were labeled on the *x*-axis. n.s., $P > 0.05$; *, $P \leq 0.05$; **, $P < 0.01$; ***, $P < 0.001$.

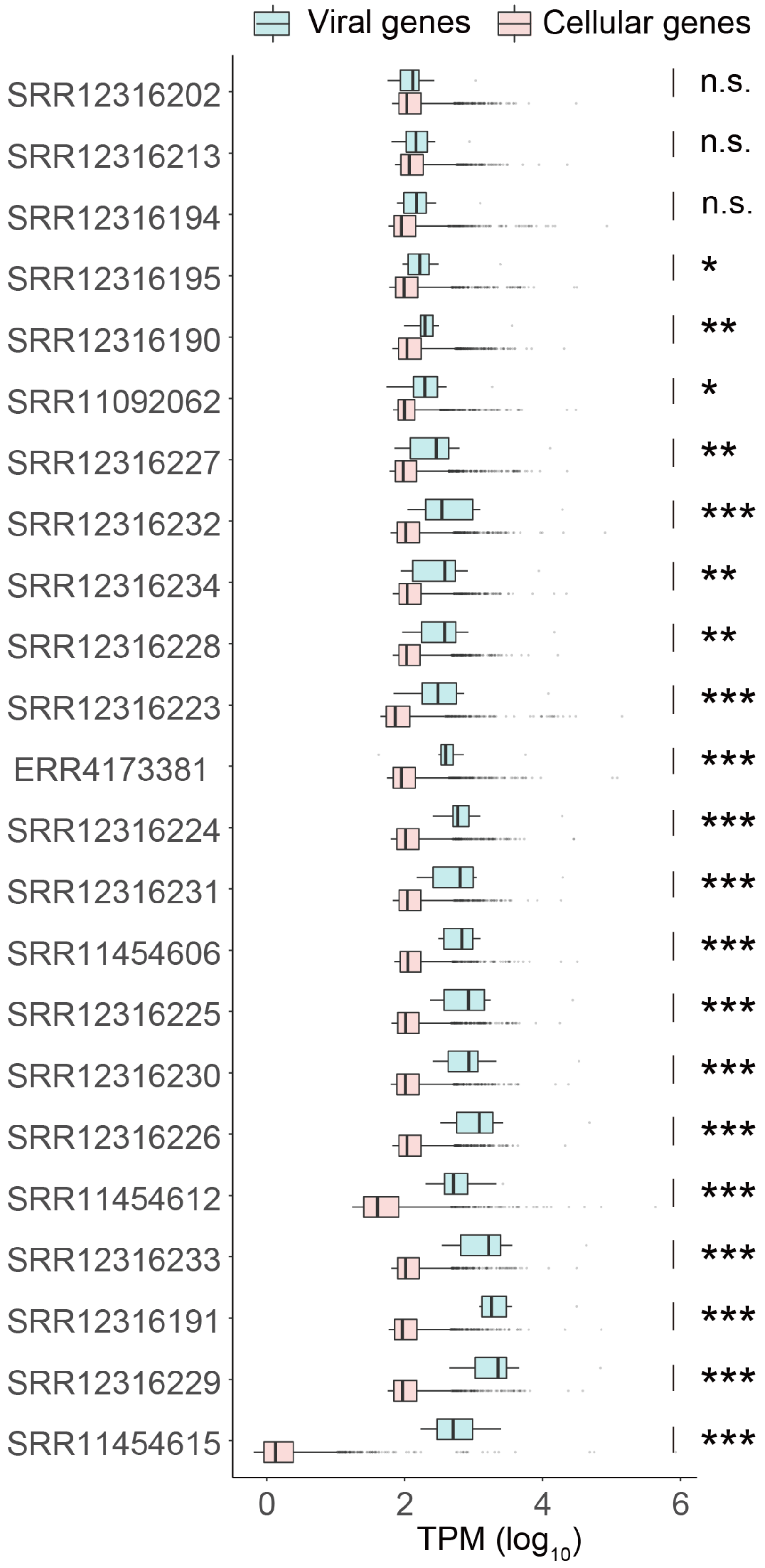

Figure S10. The gene expression levels of SARS-CoV-2 and human patients detected from metagenomic data. n.s., $P > 0.05$; *, $P \leq 0.05$; **, $P < 0.01$; ***, $P < 0.001$.

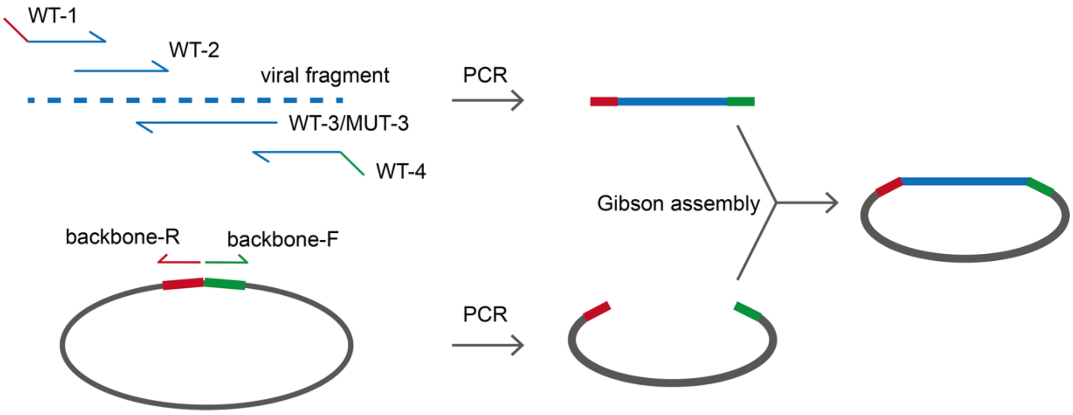

Figure S11. A scheme of the plasmid construction process. To construct viral fragments with homologous overhangs, we utilized WT-1, WT-2, WT-3 (or MUT-3), and WT-4 primers in an overlap PCR strategy. Briefly, the red or green portions on the WT-1 and WT-4 primers represent 20nt-long homologous overhangs used for connecting to the plasmid backbone. WT-1 is approximately 75nt, while WT-2, WT-3, MUT-3, and WT-4 are approximately 87nt. The specific length of the primers may vary slightly when constructing different synonymous mutation vectors. The viral segment synthesis did not use a template sequence, but was generated directly through two stages of PCR. The first stage involved amplification using the WT-2 and WT-3 primer pair, which contain approximately 17nt of complementary homologous sequence at their 3' ends. The second stage involved the addition of the WT-1 and WT-4 primers to the product of the first stage, with these primers containing approximately 17nt of homologous sequence with WT-2 and WT-3, respectively. The first stage involved 5 PCR cycles, while the second stage involved 25 PCR cycles. The final product of this process is a wild-type viral sequence fragment with homologous arms on both sides. MUT-3 and WT-3 differ by a single nucleotide, which can be used to construct a mutant viral sequence fragment following the same process as for the wild-type. We obtained the complete viral fragment by gel recovery. Subsequently, we employed Gibson assembly to ligate the products of the two processes.
